# Preparation and Degradation Performance Study of P(AM/GG/PEGDA) Nanocomposite Self-Degradation Gel Plugging Material

**DOI:** 10.3390/gels9090735

**Published:** 2023-09-09

**Authors:** Dan Bao, Siyuan Liu, Xianli Zhang, Feng Li, Jiaqin Wang, Huan Jia, Shanghao Liu, Peng Zhang

**Affiliations:** 1 School of Chemistry and Chemical Engineering, Chongqing University of Science and Technology, Chongqing 401331, China; liusyb1ack@foxmail.com (S.L.); 2021205173@cqust.edu.cn (X.Z.); 2022205018@cqust.edu.cn (J.W.); 2022205066@cqust.edu.cn (S.L.); zhangpengchina@foxmail.com (P.Z.); 2 Chongqing Oil and Gas Chemical Engineering Technology Research Center, Chongqing 401331, China; 3 Gree Energy Services Inc., Shenzhen 518000, China; feng.li@gree-oilgas.com (F.L.); jeffrey.jia@gree-oilgas.com (H.J.)

**Keywords:** reservoir, drilling fluid lost circulation, self-degradation plugging material, gel, degradation factor

## Abstract

Lost circulation is a world-class problem, and the contradiction between plugging and unplugging in reservoirs is a problem that needs to be solved urgently. The traditional LCM is not suitable for reservoirs and the complex subsequent operations. Currently, a self-degrading plugging material is proposed. In this paper, a new self-degradation plugging material, CKS-DPPG, was prepared by AM, GG, nano silica, and PEGDA. The effects of reactant concentration, pH, mineralization, etc., on the swelling and degradation performance of CKS-DPPG were investigated. The plugging capacity was tested by fracture plugging equipment, and the mechanism of self-degradation was revealed. The results show that the CKS-DPPG reached a 50% degradation rate in 54 h and complete degradation in 106 h at 80 °C and pH = 8. Low temperatures, high mineralization, and weak alkaline conditions prolong the complete degradation time of CKS-DPPG, which facilitates subsequent operations. The simulation of the 3 mm opening fracture plugging experiment showed that the pressure-bearing capacity reached 6.85 MPa and that a 0.16 MPa pressure difference could unplug after degradation. The ester bond of PEGDA is hydrolyzed under high-temperature conditions, and the spatial three-dimensional structure of CKS-DPPG becomes linear. The CKS-DPPG can effectively reduce subsequent unplugging operations and lower production costs.

## 1. Introduction

Lost circulation refers to the phenomenon that drilling fluid leaks into the formation through fracture caused by the pressure of the drilling fluid being higher than the formation pressure during the drilling process, including permeable lost circulation, fractural lost circulation, and cavernous lost circulation. Lost circulation may result in matrix permeability decreasing, oil and gas channels becoming choked, non-production time (NPT) increasing, etc. [1,2], and in severe cases, even secondary reservoir contamination and well collapse [3,4,5]. The conventional countermeasures use lost circulation materials (LCM) to prevent and treat leakage. Nowadays, those LCMs include bridging materials [6,7,8], high-filtration materials [9,10,11], and polymer materials [12,13,14]. Compared with other formations, reservoir fractures may lead to leakage of drilling fluid during well workover operations. They can improve recovery efficiency during subsequent oil and gas recovery operations. Due to the weak physical properties of the reservoir, traditional high-strength plugging materials may cause irreversible damage to the reservoir, and subsequent unblocking operations are cumbersome; therefore, they are unsuitable to be used in the reservoir.

The concept of a temporary plugging agent (TPA), which can plug the fractures to prevent lost circulation during the operations of well workover and dissolve and unblock the fracture after well completion, is proposed. When injected into the formation with drilling fluid, TPA will seal the fractures during the operations of well workover and dissolve and unblock under certain conditions after well completion, with the consequence of not affecting the gas and oil recovery efficiency. According to the different soluble conditions, TPA can be divided into three types: acid-soluble TPA, oil soluble TPA, and water-soluble TPA. Acid-soluble TPA mainly contains cellulose, calcium carbonate, etc. What causes the consequence of limited plugging ability is the poor strength of cellulose and the terrible adaptability of calcium carbonate, and an additional acid solution shall be injected to dissolve TPA after well completion, which may cause contamination of the reservoir [15,16]. Oil-soluble TPA mainly refers to resin, which has many lipophilic groups. The resin commonly has a high physical capacity but is brittle and deforms in high-temperature conditions, which may cause mass loss and increase the sealing cost [17,18]. Water-soluble TPA mainly refers to those polymers that are composed of natural biodegradable material and have the advantages of good compatibility with drilling fluid and less damage to the environment compared with others. Conversely, the disadvantage of water-soluble TPA is that it is poor in stability and physical properties in high-temperature high-pressure (HTHP) [19,20].

Based on the above, a new plugging material named self-degradable pre-formed plugging gel (s-DPPG), which can degrade and unblock the fractures after well completion spontaneously, is supposed to be developed. It is currently reported that introducing hydrolyzable long molecular chains or crosslinkers achieves degradation spontaneously. The hydrolysis reaction of ester bonds is reversible. Under the high temperature and alkaline conditions of the reservoir, the hydrolysis of ester bonds is promoted, and the reaction between the generated carboxylic acid and the OH^−^ in the environment continues to promote the hydrolysis reaction. The “bridge” connecting different molecular chains breaks, and the spatial three-dimensional structure collapses into a linear structure. The repulsive force between molecular chains dominates as the distance between molecular chains increases and finally unblocks under pressure differences. Xiong et al. [21] used polylactic acid (PLA), polyglycolic acid (PGA), polybutylene succinate (PBS), etc. prepolymers as row materials to prepare a polyester material whose degradation rate can reach 99% at 150 °C. Furthermore, the acidic environment can accelerate the process of degradation. Li et al. [22] used PLA, acrylamide (AM), and N,N-Methylenebis(2-propenamide) (MBA) as row materials to prepare the degradable temporary material TZDJ with a core-shell structure. When the shell, which acts as the sacrificial agent and the shielding agent, is hydrolyzed, the core of PLA is exposed to the environment and attacked by water molecules, leading to hydrolysis with the consequence of unblocking the fractures. On the other hand, to achieve degradation, you can also introduce hydrolyzable crosslinkers. Zheng et al. [23] used AM and acrylic acid (AA) as polymeric monomers and an azo-type thermosensitive crosslinker, which is prepared from azobisisobutyramidine hydrochloride (V-50) and maleic anhydride, to prepare a thermosensitive self-degradable gel that is more stable in HTHP compared with conventional TPA and degrades completely within 5 h at 100 °C. Additionally, high temperatures, an alkaline/acid environment, etc. can efficiently accelerate degradation. Zhang et al. [24] used AM and 2-Acrylamido-2-methyl-1-propane sulfonic acid (AMPS) as polymeric monomers and polyethylene glycol diacrylate (PEGDA) as the crosslinker to prepare a self-degradable gel material. A series of experiments was carried out to investigate the influence of the molecular weight of PEGDA on the performance of self-degradable gel and found that when the molecular weight of PEGDA decreased, the stability and hydrolysis time of gel increased, and the swelling volume rate decreased. Zhao et al. [25] investigated the influence of different component concentrations on the swelling volume rate and found that the swelling volume rate decreased with the increase in concentrations of monomer and crosslinker. Currently, the main challenges faced by s-DPPG are short degradation times and low strength. The self-degrading plugging material CSK-DPPG prepared in this paper has a complete degradation time of more than 120 h under the conditions of low temperature and high mineralization of the reservoir, which can provide sufficient time for the subsequent operation.

In this paper, a self-degradable pre-formed particle gel (CKS-DPPG) suitable for the reservoir was prepared by using AM as the monomer, PEGDA (molecular weight 200) as a cross-linker, nano-silica as rigid filler particles, and ceric ammonium nitrate as the initiator to react with guar gum to form guar gum radicals, followed by the free radical polymerization reaction. CKS-DPPG was characterized by Fourier infrared spectral analysis (FT-IR), scanning electron microscope (SEM), and thermogravimetric analyzer (TGA) for its chemical structure, microscopic morphology, and thermal stability. Then, the effects of the concentration of guar gum, nano-silica, temperature, salinity, pH, and other factors on the performance of CKS-DPPG were investigated, and the degradation reaction mechanism was analyzed. The evaluation of fracture plugging performance was also carried out.

## 2. Results and Discussion

### 2.1. Characterizations of CKS-DPPG

#### 2.1.1. FT-IR Test Analysis of CKS-DPPG

**Figure 1:**
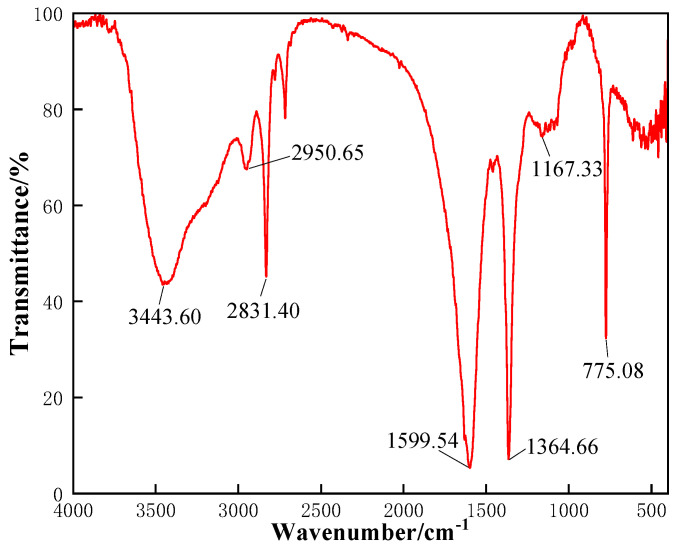
Infrared spectra of CKS-DPPG.

The FT-IR diagram is shown in Figure 1. It can be seen from Figure 1 that the absorption peak at 3443.60 cm^−1^ in the infrared image is the stretching vibration of -NH_2_ and the conjoined N-H stretching vibration at the lower wave number (3200 cm^−1^); the absorption peak at 2950.65 cm^−1^ is the saturated C-CH_2_-C antisymmetric stretching vibration because saturated C-H stretching vibration less than 3000 cm^−1^; 2831.40 cm^−1^ is aldehyde peaks, and the aldehyde peaks at 1650 cm^−1^ to 1750 cm^−1^ were covered by the peak at 1599.54 cm^−1^; the absorption peak at 1599.54 cm^−1^ is the N-H shear deformation vibration; the absorption peak at 1364.66 cm^−1^ is the C-O-C stretching vibration in the ester group; the absorption peak at 1167.33 cm^−1^ and the latter peak are the antisymmetric and symmetric stretching vibrations of C-O-C; the absorption at 775.08 cm^−1^ is a planar wobble vibration of the sub-methyl chain of the polymer. The characteristic peaks of the amide and ester groups were observed in the infrared spectrum, and the aldehyde peak from the guar gum reaction was present. Hence it was tentatively considered that the target product had been formed.

#### 2.1.2. TGA of CKS-DPPG

**Figure 2:**
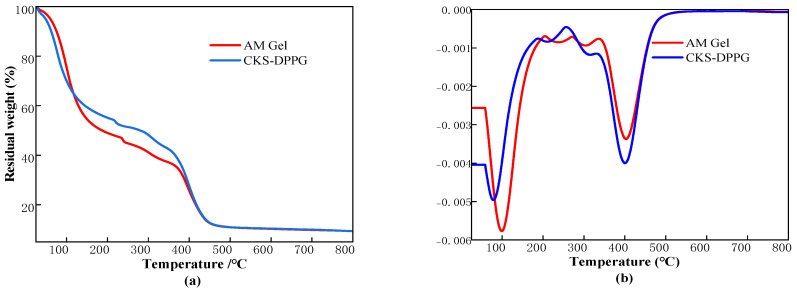
TGA (**a**) and DTG (**b**) analysis of AM Gel and CKS-DPPG.

The TGA images of s-PDDG are shown in Figure 2. It can be seen from Figure 2 that CKS-DPPG weight loss is divided into three main stages. The first stage is 25–120 °C. The mass loss in this process is mainly caused by the evaporation of water from the gel, and the rate of water evaporation is faster due to the increased pore structure and increased specific surface area in the microstructure caused by the addition of nano silica. The second stage is 120~400 °C. The mass loss in this process is mainly due to the thermal breakage of the molecular chains in the CKS-DPPG into smaller molecular chains; the third stage is after 400 °C. The mass loss in this process is due to the complete thermal degradation of the undecomposed small molecule chains and organic matter. Compared to CKS-DPPG without nano silica, introducing nano silica significantly improves the thermal stability of the gels in the second stage and does not affect final degradation. In summary, CKS-DPPG has good thermal stability.

#### 2.1.3. Micromorphology Analysis of CKS-DPPG

**Figure 3:**
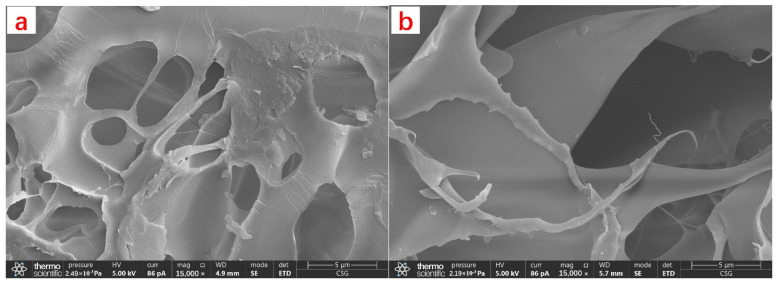

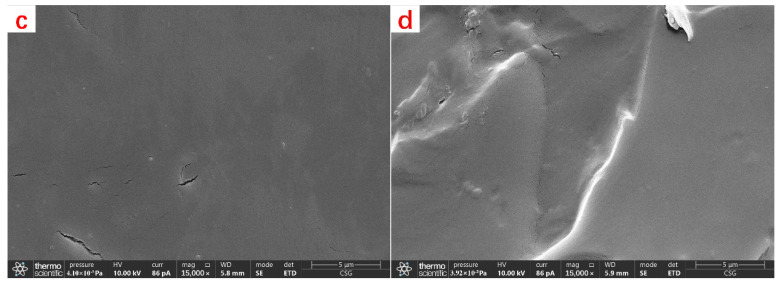
SEM images of AM Gel and CKS-DPPG. (**a**) gel without nano silica (**b**) CKS-DPPG; (**c**) the gel after degradation; (**d**) the CKS-DPPG after degradation.

SEM images of AM gel and CKS-DPPG are shown in Figure 3. Compared with Figure 3a, a reduction in microstructure cracks and an increase in porosity can be seen in Figure 3b of CKS-DPPG, which explains why water evaporation occurs at a lower temperature at the first stage after the addition of nano silica in Section 2.1.2. The SEM images of AM gel and CKS-DPPG gel after degradation are shown in Figure 3c,d. It can be clearly seen that the crosslinking sites disappear and the spatial three-dimensional structure disappears into a planar structure compared to before degradation.

### 2.2. Swelling and Degradation Properties of CKS-DPPG

**Figure 4:**
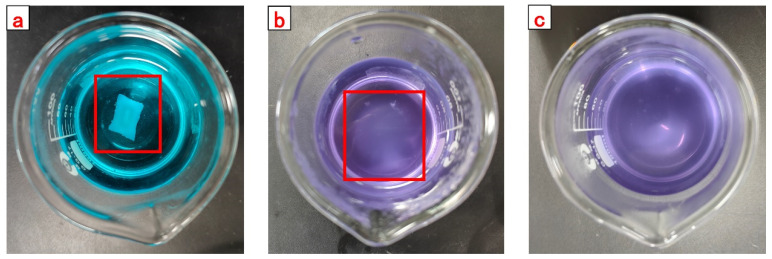
The swelling and degradation process of CKS-DPPG (methylene blue solution).

Dried CSK-DPPG was clearly visible as a white solid material when first added to the solution (methylene blue solution), as shown in Figure 4a. When the beaker was in 80 °C conditions after 48 h, the white solid material gradually became transparent, as shown in Figure 4b. After 106 h of complete degradation the gel mass disappears, and the solution becomes turbid, as shown in Figure 4c.

#### 2.2.1. Effect of Guar Gum Concentration on Swelling and Degradation Performance

**Figure 5:**
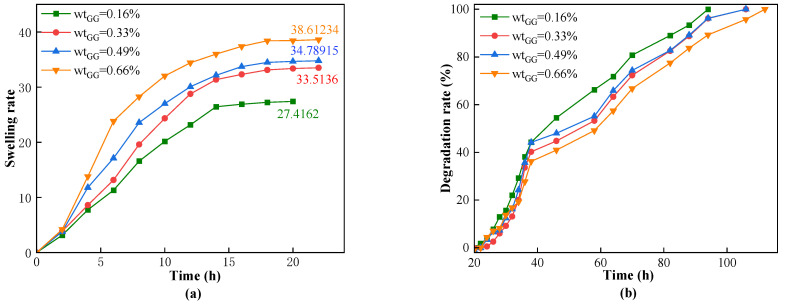
Effect of the concentration of guar gum on swelling (**a**) and degradation performance (**b**).

Guar gum is a natural polymer that we aim to introduce into the gel molecule chain to enhance the mechanical properties of the CKS-DPPG. Therefore, we designed a series of experiments with different amounts of guar gum to explore the influence of the concentration of guar gum on the swelling and degradation performance of the gel. We fixed the amounts of AM, PEGDA-200, CAN, nano-silica, and deionized water and varied the amount of guar gum (0.16%, 0.33%, 0.49%, 0.66%). When the amount of guar gum added exceeded 0.66% of the total mass, the overall viscosity of the solution increased significantly, and some of the guar gum aggregated into clusters that were insoluble in water. Excessive amounts of guar gum will also lead to a rapid increase in the viscosity of the degradation fluid, which is detrimental to the circulation of the drilling fluid. Therefore, the maximum amount of guar gum added is 0.66%. We conducted swelling and degradation tests on dry CKS-DPPG with different guar gum contents in a solution at 80 °C and pH 10, and the experimental results are shown in Figure 5.

According to Figure 5, CKS-DPPG with different amounts of guar gum reached the maximum swelling rate at around 22 h. Figure 5 also shows that increasing the amount of guar gum can increase the maximum swelling rate, with a significant improvement observed when the amount of guar gum is increased from 0.16% to 0.33%. As shown in Figure 5, CKS-DPPG with different amounts of guar gum also shows differences in the time required to reach complete degradation. The CKS-DPPG samples that achieved complete degradation first were the 0.16%, 0.33%, and 0.49% samples, with similar degradation times, while the 0.66% sample required the longest time. Our team believes that the lowest swelling rate in the 0.16% sample is due to its higher degradation rate than other samples, which is also supported by the longer time required for complete degradation. Based on this, we believe that the high-viscosity guar gum solution forms a more compact surface structure in the dried gel, making it more difficult for the solution to penetrate and resulting in fewer available OH^-^ groups in the CKS-DPPG structure. This is why the curve slope of the sample with the least amount of guar gum sharply drops after reaching the maximum swelling rate, while that of the sample with the highest amount decreases slowly. The swelling and degradation data are shown in Table 1.

Therefore, we can conclude from this section that increasing the amount of guar gum leads to an increase in the swelling rate of CKS-DPPG and a longer time for complete degradation. The performance is similar when the amount is between 0.33% and 0.49% of the solvent mass fraction; however, the group with the lower amount can effectively reduce production costs. When the amount is 0.66% of the total mass fraction, the resulting gel has a compact surface structure, which has the highest swelling rate and longest degradation time while also increasing the viscosity of the drilling fluid. We used wt_GG_ = 0.33% as the standard sample group and compared the performance with different subsequent groups. In practice, the amount of guar gum can be adjusted according to actual needs.

#### 2.2.2. Effect of Nano Silica Concentration on Swelling and Degradation Performance

**Figure 6:**
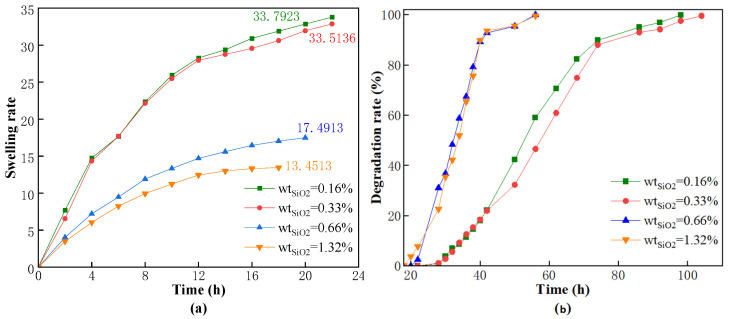
Effect of the concentration of nano silica on swelling (**a**) and degradation performance (**b**).

Nano silica which is a hydrophobic, rigid particle, was used as filler in this study. The nano silica was uniformly dispersed in a polymer base solution through stirring and then further dispersed through sonication. The polymer base solution was used to fix the nano silica in the pores of the three-dimensional structure of CKS-DPPG through a polymerization reaction. The effects of different amounts of silica dioxide (0.16%, 0.33%, 0.66%, and 1.33%) on the swelling and degradation properties of CKS-DPPG were investigated. Dry CKS-DPPG samples with different concentrations of nano silica were tested for swelling and degradation properties in a solution at pH 10 and 80 °C, as shown in Figure 6.

Figure 6 shows that increasing the concentration of nano silica leads to a decrease in the maximum swelling rate. However, when the amount is 0.33%, the difference in the maximum swelling rate is not significant compared to that of 0.16%, and it can maintain the state of maximum swelling for a longer period, which is conducive to prolonging the plugging time of CKS-DPPG in the formation. The swelling and degradation data are shown in Table 2. When the concentration of nano silica continues to increase, the maximum swelling rate decreases sharply, and the time required for complete degradation also decreases. Therefore, we believe that the addition of silica dioxide leads to an increase in the pore structure of the gel, with a greater specific surface area and more spatial cross-linking points “exposed” to the solution, increasing the probability of OH^−^ in the solution effectively crashing the gel and thus leading to faster degradation rates. The reason for the decrease in the maximum swelling rate is that when the swelling rate is constant, the hydrolysis reaction rate increases, causing the equilibrium point to shift forward. In practice, the amount of nano silica can be adjusted according to actual needs.

#### 2.2.3. Effect of Mineralization on Swelling and Degradation Performance

**Figure 7:**
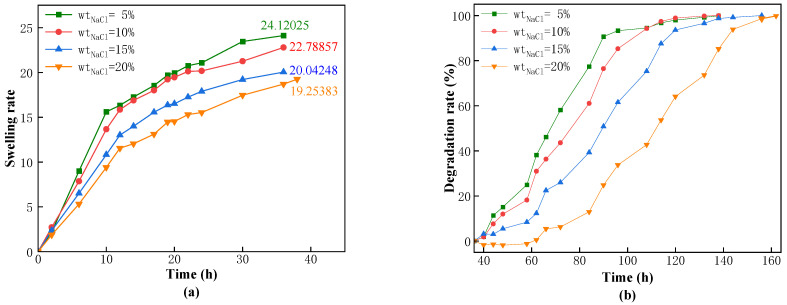
Effect of the concentration of NaCl on swelling (**a**) and degradation performance (**b**).

**Figure 8:**
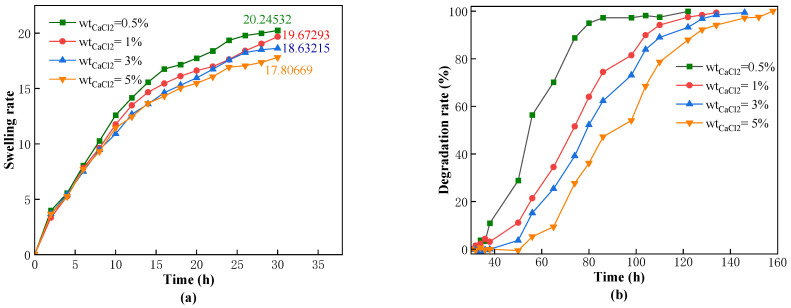
Effect of the concentration of CaCl_2_ on swelling (**a**) and degradation performance (**b**).

High concentrations of various metal ions are present in the formation of water and drilling fluids. To investigate whether the swelling and degradation performance of the CKS-DPPG in formation under high mineralization conditions would be affected, we studied the main salt ions under simulated formation conditions: Na^+^ and Ca^2+^ and their concentrations (Na^+^: 5%, 10%, 20% saturation; Ca^2+^: 0.5%, 1%, 3%, 5%) on the performance of the CKS-DPPG. The data are shown in Figure 7 and Figure 8.

**Figure 9:**
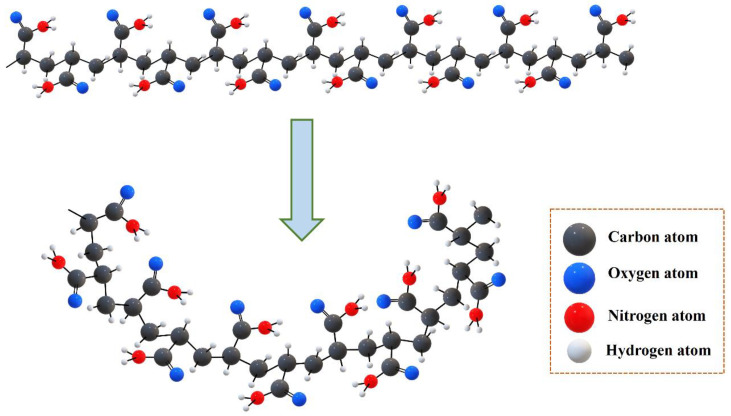
The schematic diagram of the effect of salt on molecular chains.

From Figure 7 and Figure 8, an increase in the concentration of metal ions leads to a decrease in the maximum swelling rate and an extension of the complete degradation time. The maximum swelling time increased from 12 to 42 h, and the complete degradation time extended to 162 h in a saturated sodium chloride solution environment. In addition, under the condition of equal mass fractions of sodium chloride and calcium chloride solutions, the maximum swelling multiple of CKS-DPPG in the calcium chloride solution is 19, while in the sodium chloride solution, it is 26 because the radius of the calcium ion is larger than that of the sodium ion. The swelling and degradation data are shown in Table 3 and Table 4. Our team believes that in this process: (1) the amide group is hydrolyzed into a carboxyl group under high temperature and alkaline conditions and the carboxyl group ionizes into a carboxylate anion, which makes the CKS-DPPG surface negatively charged. When there is no added metal ion, repulsion between the negative charges leads to the molecular chain’s tendency to extend. When metal ions are added, the negative charge on the surface of CKS-DPPG decreases, and the repulsion weakens. Due to intermolecular attraction, the molecular chain tends to contract, and the spatial density increases. As shown in Figure 9, water molecules are more difficult to enter the interior of CKS-DPPG, and the number of OH^−^ that can launch effective attacks decreases, increasing the time required for complete hydrolysis [26,27,28,29]. (2) The addition of metal ions leads to more counter ions entering the adsorption layer, which reduces the thickness of the double electric layer structure on the surface of CKS-DPPG and reduces the electric potential ξ. The repulsion between different molecular chains is weakened, the distance between molecular chains is reduced, and water molecules are more difficult to enter CKS-DPPG. The number of OH^−^ that can launch effective attacks decreases, resulting in an increase in the time required for complete hydrolysis. (3) The increase in the concentration of metal ions in the solution leads to a decrease in the osmotic pressure difference between the inside of CKS-DPPG and the solution, and the driving force for the solution to enter the inside of CKS-DPPG is weakened. In summary, a certain concentration of salt solution has little effect on the swelling and degradation properties of CKS-DPPG, indicating that CKS-DPPG has good adaptability to salt solutions. 

#### 2.2.4. Effect of pH on Swelling and Degradation Performance

**Figure 10:**
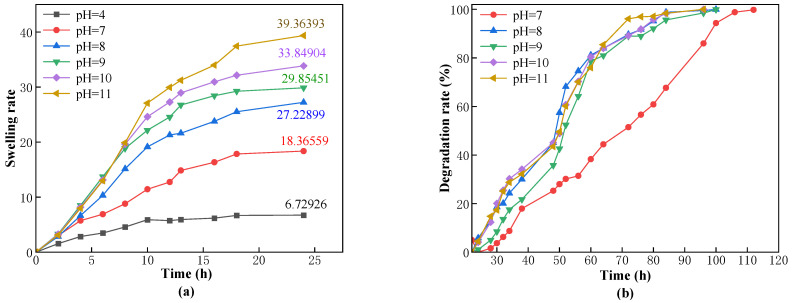
Effect of the pH on swelling (**a**) and degradation performance (**b**).

To investigate the swelling and degradation performance of CKS-DPPG under different pH conditions, we conducted tests at pH 4, 7, 8, 9, 10, and 11. The results are shown in Figure 10.

**Figure 11:**
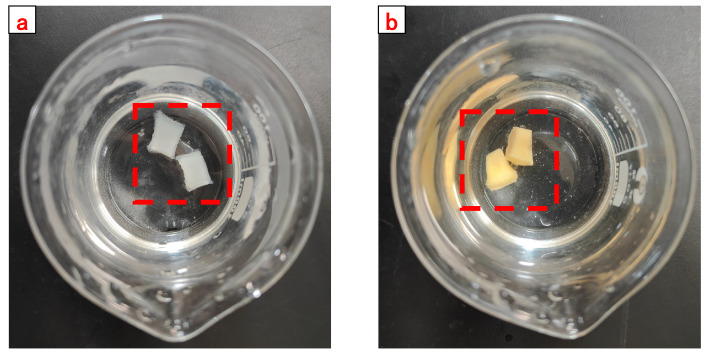
CKS-DPPG in acid solution (**a**) the initial image (**b**) 25 d later.

The amino groups on the polyacrylamide molecular chain can be partially hydrolyzed to carboxyl groups under high-temperature conditions, and the carboxyl groups ionize into carboxylate anions in solution, resulting in the negatively charged surface of CKS-DPPG. From Figure 10, under acidic conditions, CKS-DPPG initially grows rapidly in mass under the influence of osmotic pressure and then approaches a stable state with almost no further mass increase; however, the color gradually changes from white to yellow, as shown in Figure 11. Therefore, the pH = 4 data are not shown in Figure 10b and Table 5. We believe that the stable stage is due to the presence of mainly H^+^ ions in the acidic solution, which inhibits the hydrolysis of carboxyl groups. H^+^ ions have a very small radius and can easily enter the adsorption layer on the surface of CKS-DPPG, causing the electric potential ξ to decrease. At this point, the attractive force between the molecular chains predominates, and CKS-DPPG exhibits a contraction tendency. As the solution enters the interior of CKS-DPPG due to the pressure difference, the suction force increases, and the repulsive force weakens, leading to a stable overall mass when dynamic equilibrium is reached. Under neutral conditions, there are fewer free anions and cations in the water, and the overall external force on the molecular chains is smaller. The dominant force is the repulsive force between the negatively charged molecular chains; therefore, they tend to stretch out into a linear chain. At this time, the pores between the molecular chains are larger, and the solution can enter CKS-DPPG without obstruction. Under alkaline conditions, there are many OH^-^ in the solution, which diffuses into the diffuse layer and increases the thickness of the double layer, increasing the negative charge on the surface of CKS-DPPG and the repulsive force between the molecular chains. The distance between the molecular chains increases, and the solution carrying OH^−^ can more easily enter CKS-DPPG. The probability of OH^−^ effectively crashing the ester bond increases, leading to the highest degradation rate of CKS-DPPG. The swelling and degradation data are shown in Table 5. Considering that the actual working environment is neutral or weakly alkaline, CKS-DPPG can meet various requirements for practical operations.

#### 2.2.5. Effect of Temperature on Swelling and Degradation Performance

**Figure 12:**
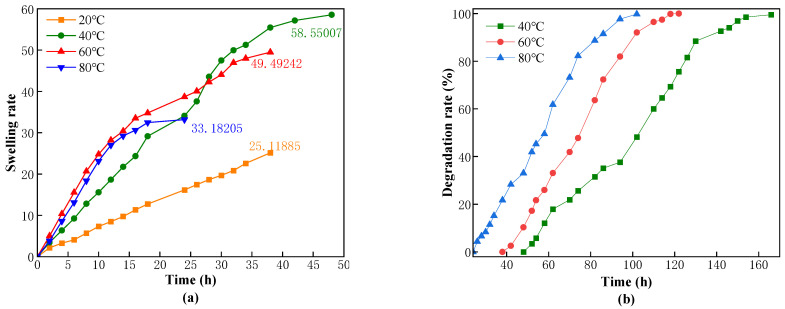
Effect of the temperature on swelling (**a**) and degradation performance (**b**).

To plug the fractures in the reservoir, the CKS-DPPG needs to have good stability under high-temperature conditions. Therefore, we simulated the effect of different temperatures on the performance of CKS-DPPG, and the experimental results are shown in Figure 12.

As shown in Figure 12, when the temperature is 20 °C, the mass of CKS-DPPG shows a linear and slow increase. Our team believes that the main reason may be that the hydrolysis of the acrylamide group into carboxylic acid is difficult to proceed under low-temperature conditions, causing the electronegativity of the gel to weaken, the repulsive force between molecular chains to decrease, the intermolecular distance to decrease, and the structure to become more compact. Therefore, the hydrolysis reaction of the ester bond is difficult to proceed at low temperatures; therefore, the 20 °C data are not shown in Figure 12b and Table 6. As the temperature gradually increases, the swelling rate increases, and the growth rate is similar when the temperature exceeds 60 °C. This process occurs because high-temperature conditions are conducive to the hydrolysis reaction of the ester bond, which causes the spatial three-dimensional structure of the gel to disintegrate into linear molecular chains. The distance between molecular chains gradually increases due to the repulsive force between negative charges, further promoting the hydrolysis reaction. Therefore, the 80 °C condition reached the inflection point first. The time required for the degradation of CKS-DPPG was greatly influenced by the temperature. When the temperature was reduced from 80 to 40 degrees, the degradation time was extended from 102 to 166 h. The swelling and degradation data are shown in Table 6. The reason is that the hydrolysis reaction of the ester bond is reversible, and the hydrolysis reaction of the ester bond will be promoted under high-temperature conditions, and the carboxylic acid produced by the reaction will then react with OH^−^ in the environment to further promote the reaction. In practice, we can predict the plugging time of CKS-DPPG according to the temperature.

#### 2.2.6. Effect of CKS-DPPG Size on Swelling and Degradation Performance

**Figure 13:**
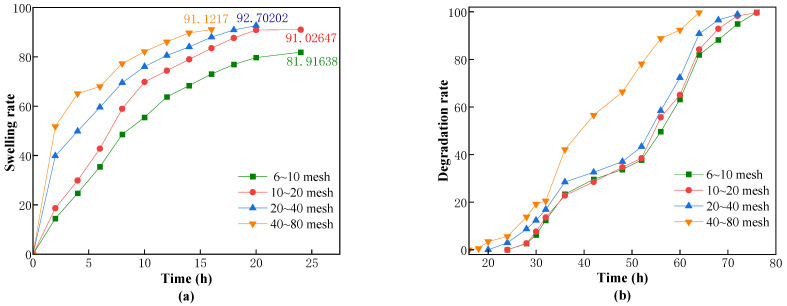
Effect of the CKS-DPPG size on swelling (**a**) and degradation performance (**b**).

To investigate the plugging performance of CKS-DPPG in reservoir fractures with different apertures, we screened CKS-DPPG of different mesh sizes (6~10/10~20/20~40/40~80 mesh) using a sieve and performed swelling and degradation tests. The experimental data are shown in Figure 13.

As shown in Figure 13, the swelling rate and the CKS-DPPG size of the mesh are positively correlated. The maximum swelling rate of CKS-DPPG at 40~80 mesh is reached in 16 h, while 6~10 mesh and 10~20 mesh take 24 h. Although both samples reach the maximum swelling rate at 24 h, the swelling rate of CKS-DPPG at 10–20 mesh is significantly higher than that of 6–10 mesh. The swelling and degradation data are shown in Table 7. The reason for this phenomenon is that the smaller the particle size at the same mass, the larger the specific surface area of CKS-DPPG and the larger the area in contact with the solution; therefore, the swelling rate will be faster. From Figure 13, the degradation rate of CKS-DPPG with the smallest particle size is significantly higher than that of the other samples, with complete degradation at 64 h. The degradation time is faster for the CKS-DPPG with smaller particle sizes; however, there is no significant difference for other samples. This may be due to the spatial stacking effect of different particle sizes of CKS-DPPG after water absorption, with large particles bridging and small particles filling, forming a dense structure, making it difficult for the solution to enter the interior of CKS-DPPG. Therefore, the difference in degradation rate is not significant. In practice, we can adjust the plugging time by compounding CKS-DPPG according to the particle size.

### 2.3. Test Results of Plugging Performance

**Figure 14:**
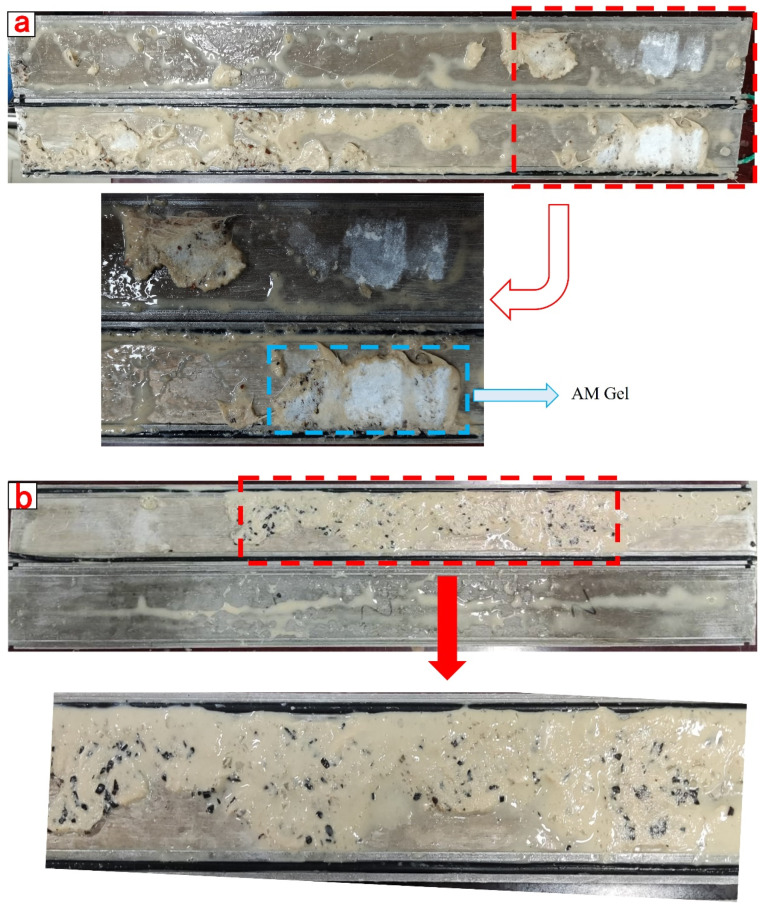
Images of AM Gel before degradation and after degradation in simulated fracture. (**a**) before degradation; (**b**) after degradation.

**Figure 15:**
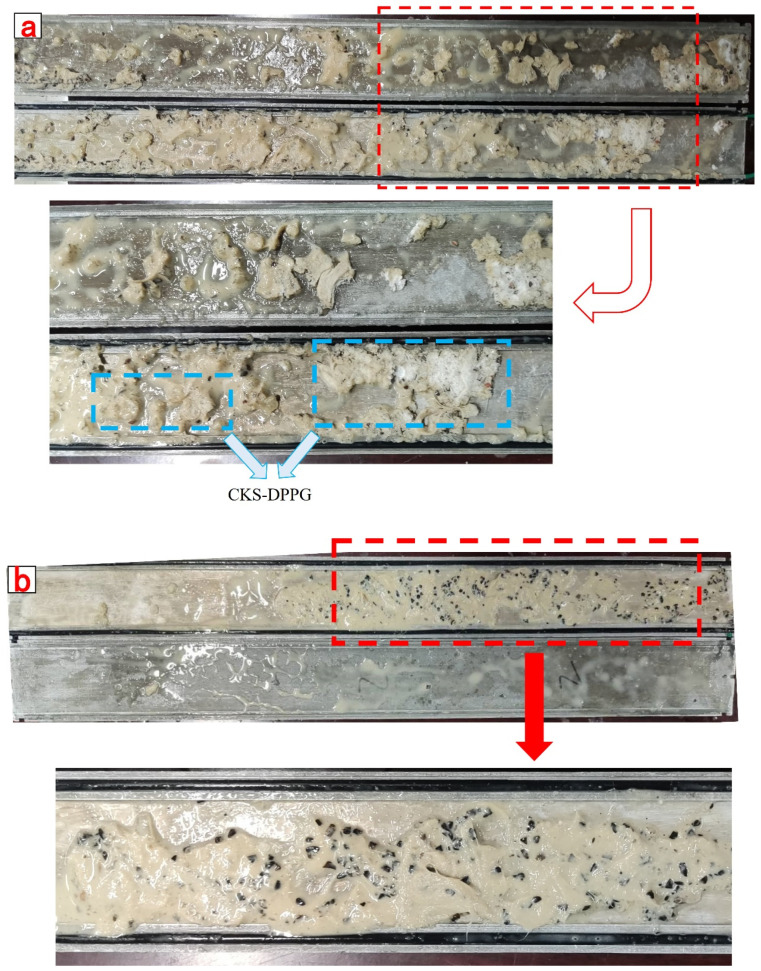
Images of CKS-DPPG before degradation and after degradation in simulated fracture. (**a**) before degradation; (**b**) after degradation.

The compounded CKS-DPPG gradually swells by absorbing water and is compressed by the pressure of drilling fluid to form a dense plugging layer in the simulated fracture, which can efficiently plug the fracture and prevent the situation of lost circulation. The plugging data are shown in Table 8. Compared with AM Gel, the plugging capacity of CSK-DPPG is significantly improved, from 4.85 MPa to 6.85 MPa, and the plugging layer is not broken. It can be inferred that the actual maximum pressure-bearing capacity should be greater than 6.85 MPa, which can complete the task of reservoir plugging. After the degradation of CKS-DPPG, the plugging performance is tested again, and the breakthrough pressure decreases sharply; therefore, it is judged that the CKS-DPPG plugging layer can be unblocked under the action of the pressure difference after self-degradation, as shown in Figure 14 and Figure 15. 

## 3. Conclusions

In this paper, a natural macromolecule, guar gum, and PEGDA as the cross-linker with ester bonds are introduced as the basis of conventional gel plugging materials and prepared as CKS-DPPG, which can degrade spontaneously in the reservoir through free radical polymerization. The following conclusions are obtained through experimental investigation and analysis:

1. The CKS-DPPG is successfully prepared with acrylamide and guar gum as monomers, PEGDA as a cross-linker, nano-silica as rigid filler particles, and ceric ammonium nitrate as an initiator. The optimal formulation is AM (30% mass fraction) + PEGDA (molecular weight 200, 0.24% mass fraction) + GG (0.33% mass fraction) + nano silica (0.33% mass fraction) + ammonium cerium (IV) nitrate (0.1% mass fraction) + deionized water. After complete degradation, the CKS-DPPG transforms liquid into solid without leaving any solid residue.
2. SEM, FT-IR, and TGA were used to characterize the CKS-DPPG and confirm the synthesis of the target product. The CKS-DPPG has good thermal stability and plugs the fractures effectively under the temperature of the reservoirs. The complete degradation time is 106 h at 80 °C and pH = 10.
3. The effects of guar gum concentration, nano silica concentration, temperature, pH, particle size, and mineralization on the performance of CKS-DPPG are investigated. It is found that the actual situation of weak alkaline and high mineralization conditions can inhibit the degradation of CKS-DPPG, and the actual reservoir conditions can effectively prolong the plugging and complete degradation time of CKS-DPPG. The degradation time is reasonably adjusted to facilitate subsequent construction.
4. Compared with AM Gel, CKS-DPPG has better pressure-bearing and plugging capacities, from 4.8 MPa to 6.85 MPa. The CKS-DPPG can efficiently plug the reservoirs and reduce the lost circulation of drilling fluid, thus reducing costs and improving efficiency. Since CKS-DPPG does not degrade under acidic conditions and the formation is usually neutral or alkaline, it can be used in a wide range of reservoirs.

## 4. Materials and Methods

### 4.1. Materials

Acrylamide (AM, AR 99%), guar gum (GG, AR 99%), polyethylene glycol diacrylate (PEGDA, molecular weight 200), Ammonium cerium (IV) nitrate (CAN, AR 99%), sodium hydroxide (AR 99%), NaCl (AR 99%), CaCl_2_ (AR 99%), and hydrochloric acid (AR 99%) were all purchased from Shanghai Macklin Biological Co., Ltd., Shanghai, China. 

AM and GG were employed as monomers, PEGDA as the crosslinker, and APS as the initiator. Deionized water was prepared in the laboratory. The frequency of the ultrasound was 40 KHz.

### 4.2. Preparation of CKS-DPPG

**Figure 16:**
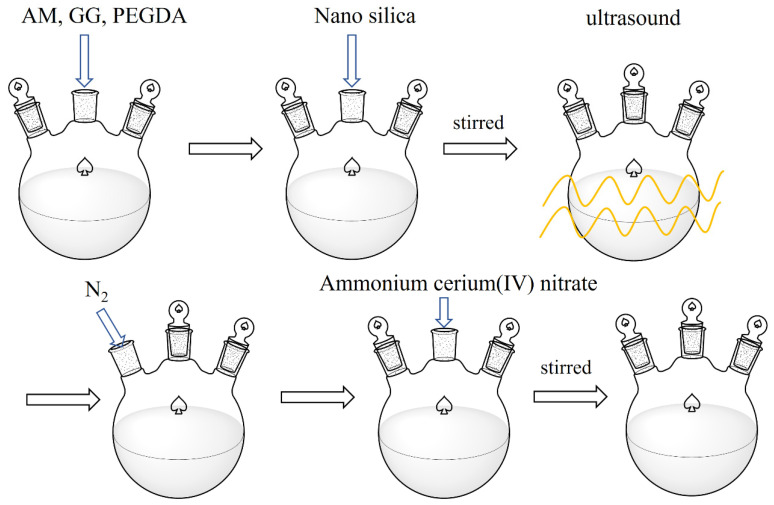
Preparation schematic of CKS-DPPG.

**Figure 17:**
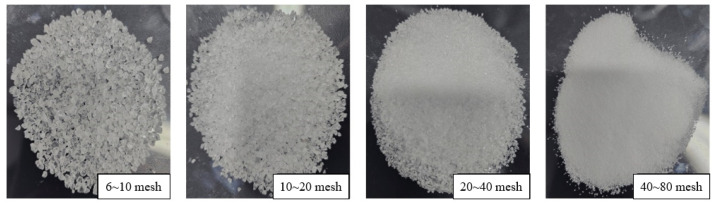
CKS-DPPG samples.

A certain amount of AM (30% mass fraction) solid powder and PEGDA (0.24% mass fraction) was weighed into a beaker filled with deionized water and mixed thoroughly with mechanical stirring for 20 min to form a homogeneous solution. A certain amount of GG (0.33% mass fraction) powder was weighed and slowly added into the solution while stirred, and the solution was guaranteed to be stirred at a constant mix speed for 30 min after the GG solid powder was completely added to form a homogeneous solution. After adding nano silica (0.33% mass fraction) and continuing mechanical stirring for 10 min, the homogeneous polymer base solution was obtained by ultrasonic dispersion treatment for 10 min. Nitrogen was introduced into the beaker, which was sealed with cling film for 20 min to remove the original oxygen. Then, a certain amount of Ammonium cerium (IV) nitrate (0.1% mass fraction) was added into a beaker in an ice water bath and stirred to form a homogeneous solution, and then the beaker was transferred to a water bath at 40 °C for a full reaction of at least 6 h. The preparation process is shown in Figure 16. With the completion of the reaction in the beaker, the gel was fished out and rinsed with acetone. The rinsed gel was transferred into the oven to deprive the body of water and then crushed to obtain a self-degradable plugging particle gel. The product images are shown in Figure 17. The reaction mechanism is shown in Figure 1. The optimal formulation is AM (30% mass fraction) + PEGDA (molecular weight 200, 0.24% mass fraction) + GG (0.33% mass fraction) + nano silica (0.33% mass fraction) + ammonium cerium (IV) nitrate (0.1% mass fraction) + deionized water.

### 4.3. Reaction Mechanism

**Figure 18:**
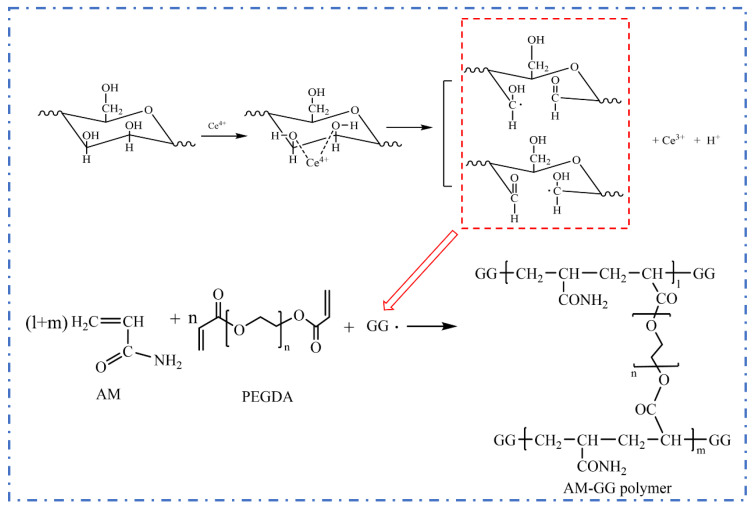
The reaction mechanism of preparation CKS-DPPG.

The adjacent double hydroxyl in guar gum is oxidized by the cerium ion (IV) to form guar gum radicals. Guar gum radicals as initiators induce free radical polymerization of acrylamide monomer and PEGDA crosslinker to produce AM-GG polymer. The reaction mechanism is shown in Figure 18.

### 4.4. Characterization of CKS-DPPG

After being placed into a vacuum oven for 1 d, the CKS-DPPG particles prepared in Section 4.2 were ground into 200–300 mesh powder and mixed with dry KBr homogeneously, then pressed into sheets under 20 MPa pressure by a tablet press, which would be used to be tested by a Fourier transform infrared spectrometer.

The pre-degradation CKS-DPPG was lyophilized and then gold-plated before being morphologically characterized using SEM. The CKS-DPPG was degraded for 24 h, then lyophilized and gold plated, and the gels were characterized using SEM.

The formation temperature where CKS-DPPG works is often high, which requires CKS-DPPG to have better thermal stability. The dried sample was heated to 800 °C at a heating rate of 20 °C/min in a nitrogen purge environment, and the mass change curve was recorded.

### 4.5. Measurement of Swelling and Degradation Properties of CKS-DPPG

The swelling and degradation processes of the dried CKS-DPPG in solution are simultaneous. The swelling phase is considered when the swelling rate is greater than the degradation rate, and vice versa for the degradation phase. Since the edges of CKS-DPPG are gradually degrading during the process of degradation, the traditional method of weighing the reduction of mass may lead to a large error due to the inability to accurately weigh the softened layer at the edges. Therefore, the swelling rate (*η_s_*) test is carried out by placing the dry CKS-DPPG (*m*_0_) into a pocket in which small molecule chains can pass and then weighing the mass change of the pocket. The pockets are placed in solutions from different environments to investigate the effects of the concentration of guar gum, the concentration of nano silica, salinity, pH value, temperature, and particle size on the swelling and degradation properties. The pockets are completely submerged in the solution, and then the pockets are removed at certain time intervals. The surface of the pockets is dried up and weighed, and the mass of that moment is recorded (*m_t_*). The swelling rate is calculated according to Equation (1), and the swelling rate is considered to have reached a maximum when the mass starts to stabilize or decrease.

(1) $$\eta_{s}=\frac{m_{t}-m_{0}-m_{w}}{m_{0}}$$

where $\eta_{s}$ is the swelling rate; *m_t_* is the mass of CKS-DPPG at *t* moment; *m*_0_ is the mass of initial dry CKS-DPPG; *m_w_* is the mass of wet pocket.

The degradation process starts to be calculated when the swelling rate reaches its maximum. The mass of the pockets (*m_t_*) is weighed at certain time intervals, and the degradation is considered complete when there is no obvious residue in the pockets and the mass is stable. The swelling rate is calculated according to Equation (2).

(2) $$\eta_{d}=\frac{m_{max}-m_{t}}{m_{max}-m_{w}}*100\%$$

where $\eta_{d}$ is the degradation rate; *m_max_* is the max mass of CKS-DPPG; *m_t_* is the mass of CKS-DPPG at *t* moment; and *m*_0_ is the mass of initial dry CKS-DPPG.

### 4.6. Plugging Property Evaluation

**Figure 19:**
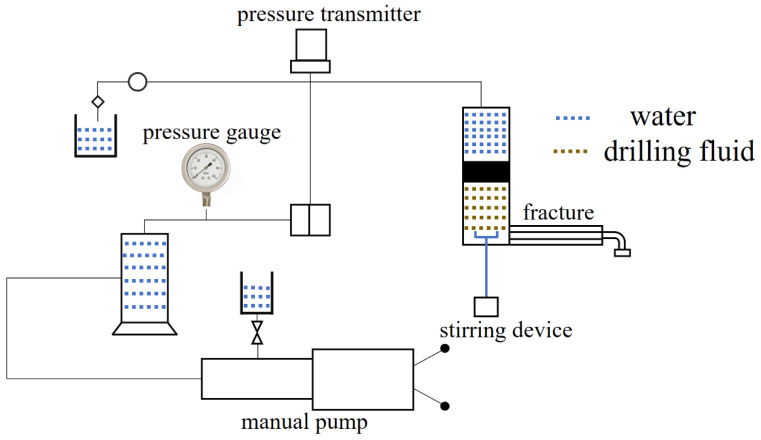
Diagram of simulated plugging equipment.

The simulated fracture plugging equipment is shown in Figure 19. Add the compound CKS-DPPG to the simulated fracture equipment and set the maximum pressure to 6 MPa with the manual pump. If the pressure reaches 6 MPa, the device will stop pressurizing. If not, then the maximum breaching pressure will be recorded. Turn on the equipment, and the pressure gradually increases.

The composition of the drilling fluid is 2000 mL of slurry (3%) + 0.2% CMC-LV, +0.5% KPAM, +3% SPNH, +3% superfine calcium carbonate. The composition of the compound CKS-DPPG is 1 g CKS-DPPG (10~20 mesh) + 1 g CKS-DPPG (20~40 mesh) + 1 g CKS-DPPG (40~80 mesh) + 1.5 g CKS-DPPG (>80 mesh) + 1 g walnut shell (10~12 mesh) + 1 g walnut shell (12~16 mesh) + 1 g quartz (12~16 mesh) + 1 g superfine calcium carbonate + 0.5 g fiber.

## Figures and Tables

**Table 1:** Data on swelling and degradation at different guar gum concentrations.

Mass Fraction of GG	Max Swelling Rate Time/h	Max Swelling Multiple	50% Degradation Time/h	Complete Degradation Time/h
0.16%	20	27.4162	44	94
0.33% (Standard sample)	22	33.5136	54	106
0.49%	22	34.78915	56	106
0.66%	22	38.61234	58	112

**Table 2:** Data on swelling and degradation at different nano silica concentrations.

Mass Fraction of Nano Silica	Max Swelling Rate Time/h	Max Swelling Multiple	50% Degradation Time/h	Complete Degradation Time/h
0.16%	22	33.7923	54	94
0.33% (Standard sample)	22	33.5136	54	106
0.66%	20	17.4913	34	52
1.32%	18	13.4513	34	52

**Table 3:** Data on swelling and degradation at different NaCl concentrations.

The Concentration of NaCl	Max Swelling Rate Time/h	Max Swelling Multiple	50% Degradation Time/h	Complete Degradation Time/h
Standard Sample	22	33.5136	54	106
5%	36	24.1202	68	132
10%	36	22.7885	78	138
20%	36	20.0424	90	156
Saturated	38	19.2538	112	162

**Table 4:** Data on swelling and degradation at different CaCl_2_ concentrations.

The Concentration of CaCl_2_	Max Swelling Rate Time/h	Max Swelling Multiple	50% Degradation Time/h	Complete Degradation Time/h
Standard Sample	22	33.5136	54	106
0.5%	30	20.24532	54	122
1%	30	19.67293	74	134
3%	30	18.63215	78	146
5%	30	17.80669	92	158

**Table 5:** Data on swelling and degradation at different pH conditions.

pH	Max Swelling Rate Time/h	Max Swelling Multiple	50% Degradation Time/h	Complete Degradation Time/h
7	24	18.36559	72	112
8	24	27.22899	49	100
9	24	29.85451	49	100
10 (Standard sample)	24	33.84904	50	96
11	24	39.36393	50	96

**Table 6:** Data on swelling and degradation at different temperatures.

Temperature/°C	Max Swelling Rate Time/h	Max Swelling Multiple	50% Degradation Time/h	Complete Degradation Time/h
40	48	58.55	106	166
60	38	49.4924	78	122
80 (Standard sample)	24	33.182	58	102

**Table 7:** Data on swelling and degradation at different particle sizes.

Particle Size/Mesh	Max Swelling Rate Time/h	Max Swelling Multiple	50% Degradation Time/h	Complete Degradation Time/h
Standard sample	22	33.5136	54	106
6–10	24	81.91638	56	76
10–20	24	91.02647	54	76
20–40	20	92.70202	54	72
40–80	16	91.1217	40	64

**Table 8:** Table of plugging capacities of different materials.

Sample		Maximum Plugging Pressure/MPa
AM Gel	Before degradation	4.85
	After degradation	0.13
CKS-DPPG	Before degradation	6.8
	After degradation	0.16

## Data Availability

The raw/processed data required to reproduce these findings cannot be shared at this time as the data also forms part of an ongoing study.
